# Metabolic comparison of aerial and submerged mycelia formed in the liquid surface culture of *Cordyceps militaris*

**DOI:** 10.1002/mbo3.836

**Published:** 2019-03-28

**Authors:** Ahmad Suparmin, Tatsuya Kato, Hiroyuki Takemoto, Enoch Y. Park

**Affiliations:** ^1^ Department of Bioscience, Graduate School of Science and Technology Shizuoka University Suruga‐ku, Shizuoka Japan; ^2^ Laboratory of Biotechnology, Green Chemistry Research Division Research Institute of Green Science and Technology, Shizuoka University Suruga‐ku, Shizuoka Japan; ^3^ Instrumental Research Support Office, Department of Bioscience, Graduate School of Science and Technology Shizuoka University Suruga‐ku, Shizuoka Japan

**Keywords:** aerial mycelia, *Cordyceps militaris*, heme biosynthesis, hypoxia, liquid surface culture, submerged mycelia

## Abstract

An entomopathogenic fungus, *Cordyceps* sp. has been known to produce cordycepin which is a purine nucleoside antimetabolite and antibiotic with potential anticancer, antioxidant and anti‐inflammatory activities. Interestingly, *Cordyceps militaris *produces significantly higher amount in a liquid surface culture than in a submerged culture. The liquid surface culture consists of mycelia growing into the air (aerial mycelia) and mycelia growing toward the bottom into the medium (submerged mycelia). In this study, to clarify roles of aerial and submerged mycelia of *C. militaris* in the cordycepin production the difference in metabolism between these mycelia was investigated. From transcriptomic analyses of the aerial and submerged mycelia at the culture of 5, 12 and 19 days, the metabolism of the submerged mycelia switched from the oxidative phosphorylation to the fermentation pathway. This activated the pentose phosphate pathway to provide building block materials for the nucleotide biosynthetic pathway. Under hypoxic conditions, the 5‐aminolevulinic acid synthase (CCM_01504), delta‐aminolevulinic acid dehydratase (CCM_00935), coproporphyrinogen III oxidase (CCM_07483) and cytochrome c oxidase 15 (CCM_05057) genes of heme biosynthesis were significantly upregulated. In addition, the liquid surface culture revealed that metabolite coproporhyrinogen III and glycine, the product and precursor of heme, were increased at 12th day and decreased at 19th day, respectively. These results indicate that the submerged mycelia induce the activation of iron acquisition, the ergosterol biosynthetic pathway, and the iron cluster genes of cordycepin biosynthesis in a hypoxic condition. Even though, the expression of the cluster genes of cordycepin biosynthesis was not significantly different in both types of mycelia.

## 1 INTRODUCTION

*Cordyceps* species were known to be the superior producers of the pharmaceutical compound and anticancer agent cordycepin (Cui et al., 2018; Cunningham, 1950; Nakamura, Yoshikawa, & Yamaguchi, 2006; Yong et al., 2018). This fungus uses a clever mechanism to infect and manipulate the internal environment of the insect immunity and resist the defenses of the insect, and it finally develops into a hyphal formation and emerges from the body of the insect (Anderson & May, 1982; Frank, 1996; Lovett & Leger, 2015). In the laboratory, hyphae formation and differentiation to the fruiting body of *C. militaris *only formed on solid media but not in liquid culture (Xiong, Xia, Zheng, Shi, & Wang, 2010). When this fungus is inoculated in a liquid surface culture (static culture), the submerged mycelia grow toward the bottom into the medium, and the hypha on the surface of the culture grows into the air and form aerial mycelia after a period of culture. This phenomenon has not been reported in the liquid surface culture of *C. militaris*. The aerial mycelia in the liquid surface culture probably produce more spores and conidia due to the hydrophobic thin layer between the medium and air. The submerged mycelia, which were in direct contact with the media, might contribute to the production and secretion of cordycepin into the media.

Previously, we reported an RNA sequencing (RNA‐Seq) analysis of differentially expressed genes (DEGs) between liquid surface and submerged cultures of *C. militaris.* Surprisingly, the analysis revealed that cordycepin was produced significantly higher in the liquid surface culture than in the submerged culture (Suparmin, Kato, Dohra, & Park, 2017). SAICAR synthase (CCM_04437) and some oxidoreductase activities were significantly upregulated in the liquid surface culture. The mycelia cover the culture medium and form a cake‐like mat. Following the formation of this thick layer of aerial mycelia, hypoxic conditions appeared in the submerged mycelia of the liquid surface culture (Keulen et al., 2003).

Oxygen is an essential and critical agent for fungal metabolism. However, during exposure to hypoxic conditions, the fungus upregulates some global transcriptome responses that affect hypoxia, such as the hypoxia inducible factor (HIF) (Bunn & Poyton., 1996; Grahl et al., 2011), zinc finger (Ernst & Tielker, 2009), Sre1 (Bien & Espenshade, 2010; Hughes & Espenshade, 2008) and heme biosynthesis (Chelstowska & Rytka, 1993). The fungal morphology also changes from yeast‐like and/or conidial into hyphae under hypoxic or anaerobic conditions (Dumitru, Hornby, & Nickerson, 2004; Goranov & Madhani, 2014; Lu, Su, Solis, Filler, & Liu, 2013; Zhao et al.., 2014).

However, the difference in the metabolism of either the aerial or submerged mycelia of *C. militaris *in the liquid surface culture has not been elucidated. Cordycepin is excreted into the culture media of *C. militaris* during its liquid surface culture. Thus, the clues to hypoxia and cordycepin biosynthesis might be found in the submerged mycelia. To analyze the difference in metabolism between the aerial and submerged mycelia during the liquid surface culture, a transcriptomic analysis was performed at every stage of growth in this study.

## 2 RESULTS

### 2.1 Mycelial morphology of *C. militaris *in liquid surface culture

**Figure 1:**
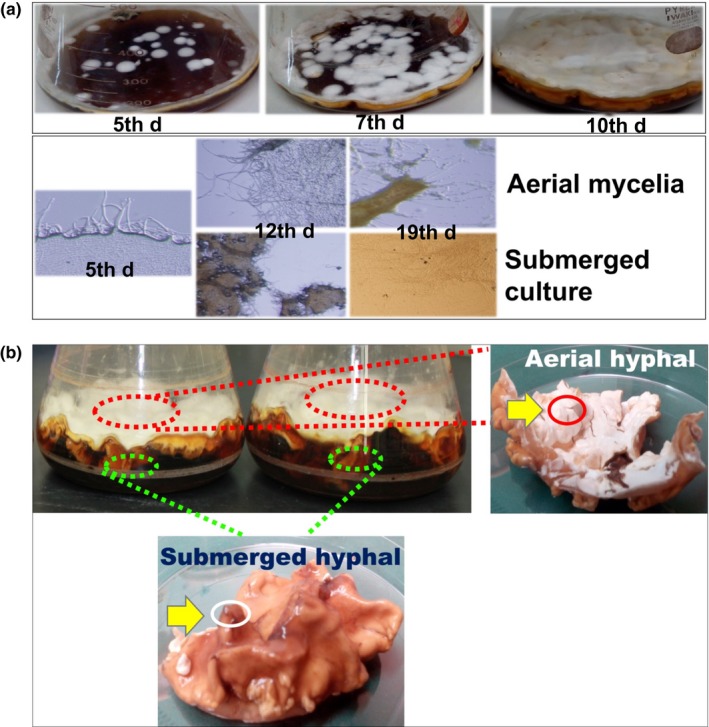
Morphology and cordycepin production of *Cordyceps militaris *in the liquid surface culture. (a) (upper panel) Floating mycelia of *C. militaris *started forming several small cake‐like mats on the 5th day of culture, partially covered the surface medium on the 7th day, and completely covered the surface medium on the 10th day (lower panel). Their morphology under the microscope indicated that the hyphae from the aerial and submerged mycelia started growing on the 5th day, developing mycelia and showed compacted mycelia on the 12th day. (b) RNA was extracted from the thick form of the aerial mycelia and the thin form of the submerged mycelia following 5, 12, and 19 days of culture

The floating mycelia of *C. militaris* started to form several small cake‐like morphologies on 5th day and did not yet cover the surface of the culture medium (Figure 1a). This is notable since small pieces of noncompartmentalized mycelia emerged spatially on the surface media. Finally, the spreading mycelia progressively stuck together during the 10 days of culture and completely covered the surface of the medium. In the beginning, the submerged hyphae were formed in the liquid surface culture of *C. militaris* prior to the hyphae growing into the air (Figure 1b). Cordycepin was produced after 11 days of culture of *C. militaris* and was only detected in the culture broth (Suparmin et al., 2017). This suggested that the cordycepin might be produced from the submerged mycelia that were soaked in the medium, after the surface of the media was covered by mycelia.

### 2.2 Overview of the differentially expressed genes (DEGs) in the aerial and submerged mycelia

The total RNA was extracted from the whole mycelia at 5th day and from the aerial and submerged mycelia at 12th and 19th day, respectively (Figure 1b). The DEGs were analyzed among the three sampling times. The transcriptome results showed that three upregulated genes were found in the aerial mycelia, e.g., *cyclin‐like F‐box *(CCM_01052, CCM_08975*, *and CCM_06327), with 7.044‐, 5.582‐, and 3.782‐fold expression, respectively, which are involved in cell fusion and hyphal anastomosis. In the submerged mycelia, two genes were upregulated with a lower fold expression level than those of the aerial mycelia, e.g., *mitochondrial fusion protein (Ugo1), *putative (CCM_07722), *HET‐C domain protein HetC *(CCM_01654) and one downregulated *membrane fusion mating protein FIG1* (CCM_01276)*,* with 4.168‐, 2.762‐, and −1.836‐fold expression, respectively (Appendix Table A1). In addition, a fusion was also involved in the mating‐type locus of the hypha, and a gene encoding a transcription factor mating‐type *MAT‐111*(CCM_06523) was only upregulated in the submerged mycelia with 3.886‐fold expression. However, the expression of the mating‐type *MAT‐1‐1‐2 *(CCM_09679) in the submerged mycelia was not significantly different. These results suggest that cell fusion and anastomosis are actively performed in the aerial mycelia to assemble the pieces of the small cake‐like mycelia. Interestingly, the homeobox transcription factor (CCM_07504) was found to be upregulated in both types of mycelia. The disruption of the homeobox gene AFLA_069100 of *A. flavus* resulted in the loss of the conidia and aflatoxin production (Cary et al., 2017). Based on the gene ontology analysis, CCM_07504 may regulate the conidiogenesis and fruiting body formation of the aerial and submerged mycelia of *C. militaris.*

**Figure 2:**
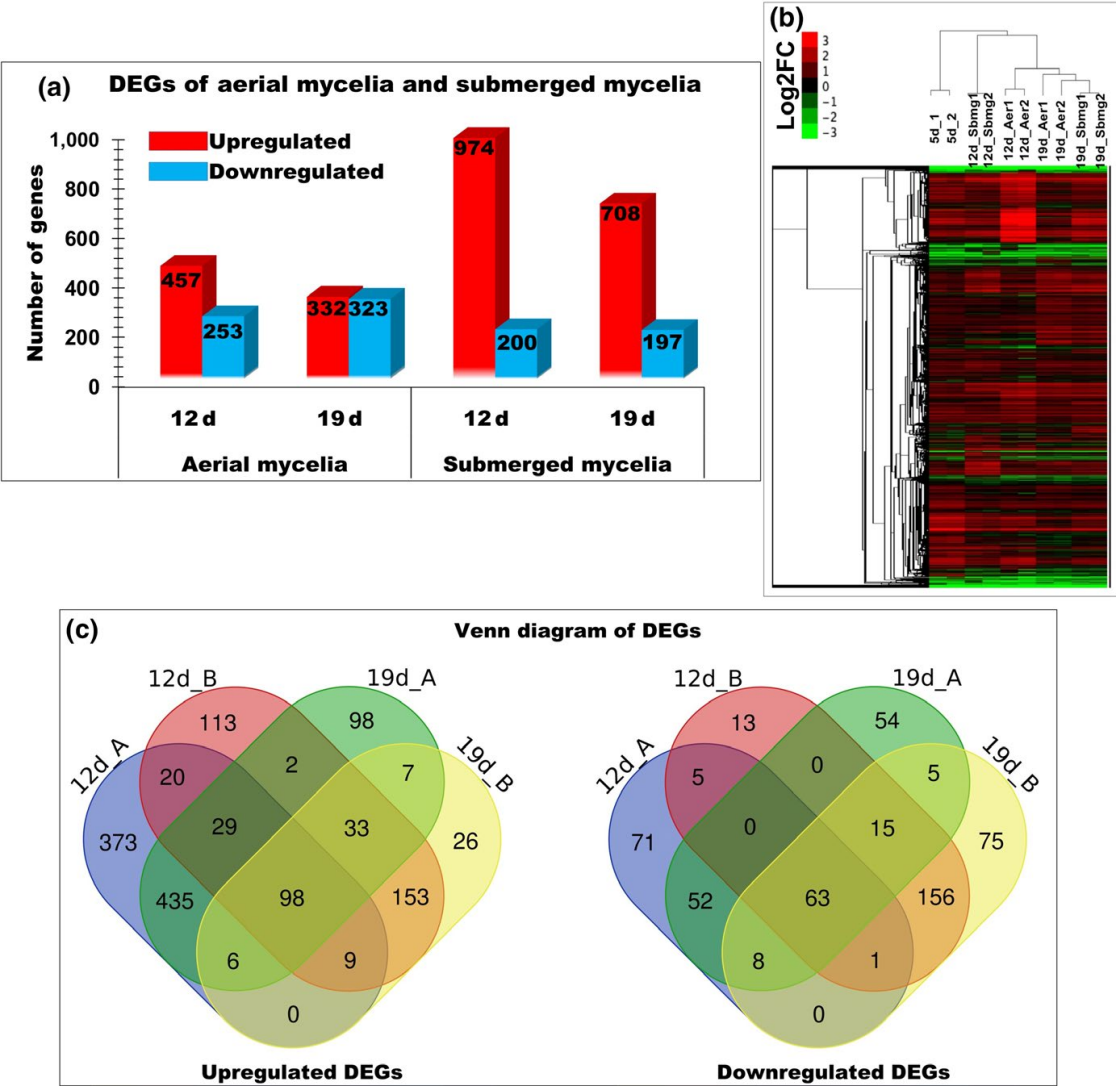
DEGs between the aerial and submerged mycelia in the liquid surface culture of *Cordyceps militaris*. (a) A total of 974 genes were upregulated (red color) in the submerged mycelia compared to the aerial mycelia, while the downregulated genes (blue color) were much lower in the submerged mycelia than in the aerial mycelia, with 332 genes significantly downregulated that kept increasing to 323 genes along with the culture periods. (b) The red color of the cluster heatmap clearly shows the significantly highest upregulated genes at 12th day of culture of the submerged mycelia. (c) Venn diagram of the upregulated and downregulated DEGs between the aerial and submerged mycelia. Approximately 98 and 63 genes were differentially upregulated and downregulated, respectively, and were maintained between both mycelia through the cultivation times

To perform transcriptomic analysis, RNA was extracted from aerial and submerged mycelia following 5, 12 and 19 days of culture. The aerial and submerged mycelia of the 5th day were used to control of the transcriptome analysis, because the mycelia began forming aerial mycelia but did not cover the surface of the medium, and cordycepin production had not yet started (Suparmin et al., 2017). The results revealed a total of 710 and 1682 DEGs using triplicate samples and a threshold set up of four‐fold change (log2 FC ≥2) and false discovery rate (FDR ≤5%) for the aerial and submerged mycelia, respectively, following 12 days of culture. In total, the upregulated genes of 974 DEGs in the submerged mycelia were higher than in the aerial mycelia. However, the downregulated genes in the aerial mycelia were much higher than in the submerged mycelia with 253 DEGs in the aerial mycelia and 200 in the submerged mycelia. In contrast, approximately 332 and 708 DEGs in the aerial mycelia and 323 and 197 in the submerged mycelia were upregulated and downregulated, respectively, following 19 days of culture (Figure 2a,b), while the expression of approximately 98 upregulated and 63 downregulated genes was maintained between both types of mycelia throughout the cultivation time (Figure 2c). The genes in the submerged mycelia still increased in activity after 19 days of culture.

**Figure 3:**
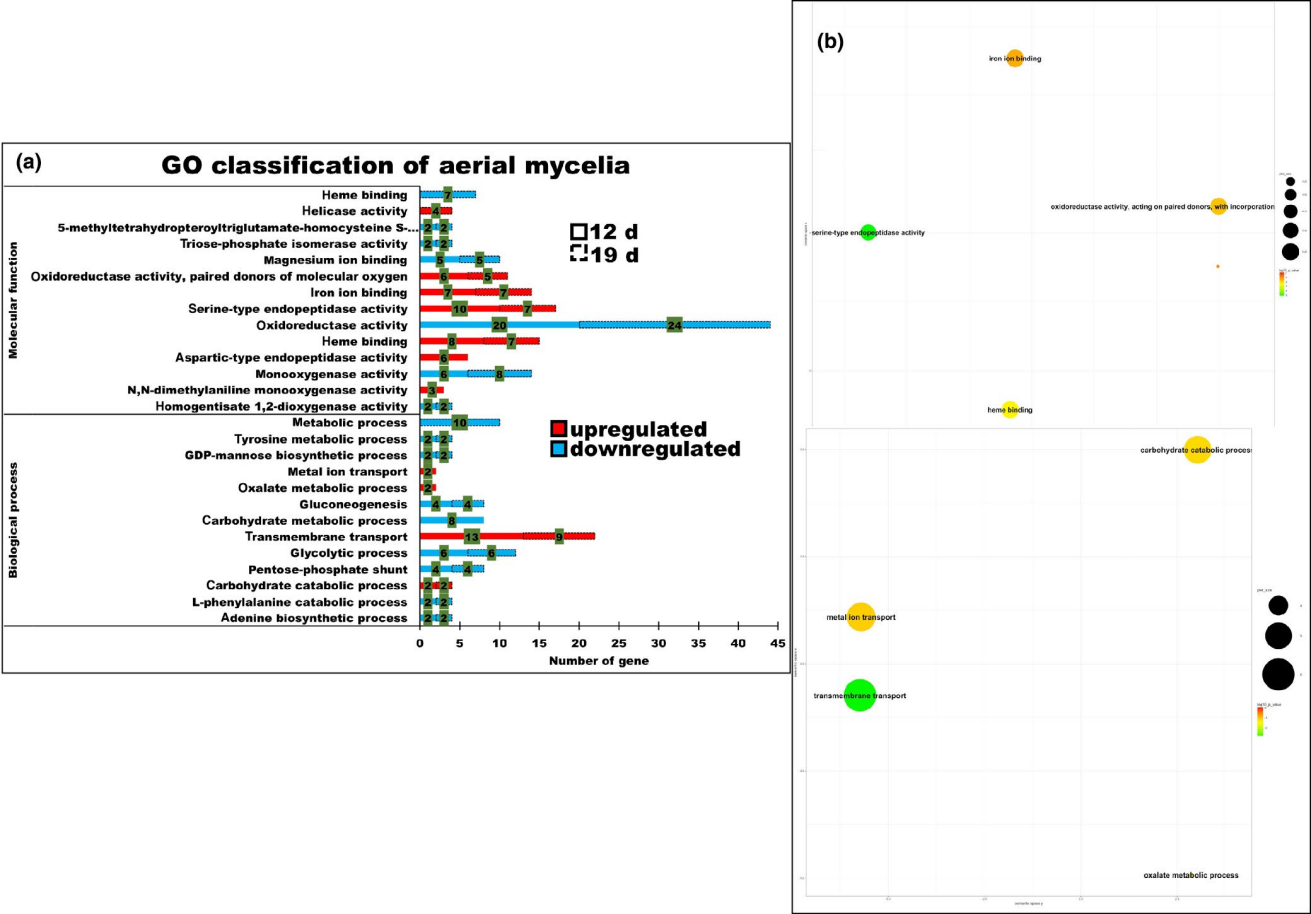
GO enrichment of the aerial mycelia. (a) The biological process terms showed that transmembrane transport was the highest proportion (numbers shown in the green bracket) of the GO enrichment, followed by the carbohydrate catabolic process either on 12th day or 19th day (dashed line), oxalate metabolism and metal ion transport only on the 12th day. While the highest proportion of the molecular function terms was a serine‐type endopeptidase, iron ion binding and oxidoreductase activity paired donors of the molecular oxygen were present through the cultivation periods. (b) Summarized results of the REViGO semantic analysis (http://revigo.irb.hr/) of the GO biological process and molecular function terms that were enriched and are represented as scatterplots in two‐dimensional space with similar GO terms indicated by the bubbles that are close together in the plot. The *p*‐value of the false discovery rates (FDR) and the GO frequency are indicated by the bubble color and bubble size. The bubbles of more general terms are larger

**Figure 4:**
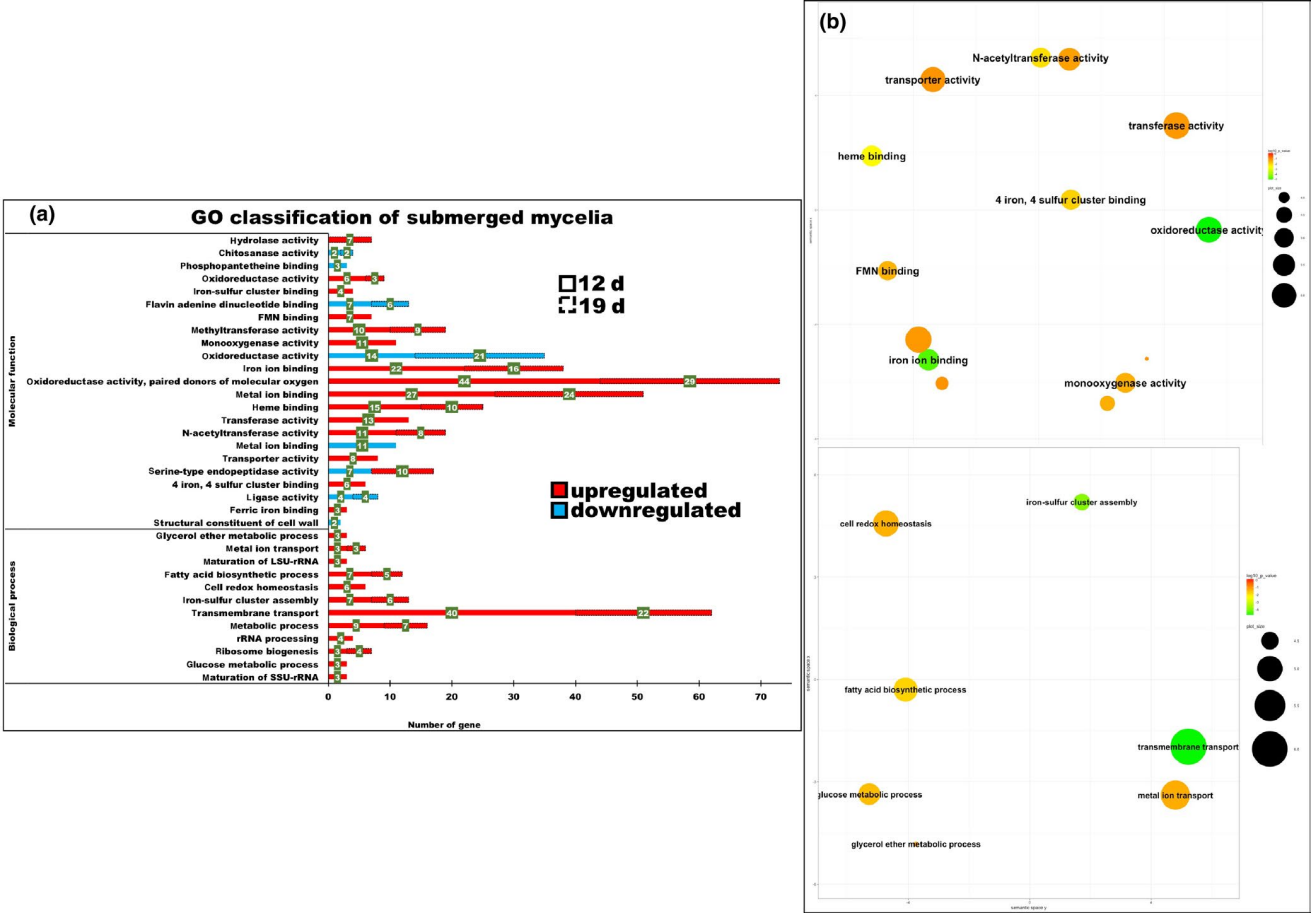
GO enrichment of the submerged mycelia. (a) The biological process terms showed that transmembrane transport was the highest proportion (numbers shown in the green bracket) of the GO enrichment, followed by metabolic process, iron‐sulfur cluster assembly, fatty acid biosynthesis, metal ion transport either on 12th day or 19th day (dashed line), and the glucose metabolic process and glycerol ether metabolic process were found only on the 12th day of the culture periods. While the highest proportion of the molecular function terms was the oxidoreductase activity, paired donors of molecular oxygen, metal ion binding, iron ion binding, heme binding and the N‐acetyltransferase activity were present through the cultivation periods. (b) Summarized results of the REViGO semantic analysis (http://revigo.irb.hr/) of the GO biological process and molecular function terms that were enriched and are represented as scatterplots in two‐dimensional space with similar GO terms indicated by the bubble form maintained close together in the plot. The *p*‐value of the false discovery rates (FDR) and the GO frequency are indicated by the bubble color and bubble size. The bubbles of more general terms are larger

The GO enrichment annotation conducted using the DAVID analysis is illustrated in Figures 3 and 4. The upregulated proportion with the molecular function throughout the culture periods among the numbers of genes was found to be higher in the aerial mycelia, such as serine‐type endopeptidase activity following 12 and 19 days of culture (10 and 7), heme binding (8 and 7), iron ion binding (7 and 7), and oxidoreductase activity particularly involved in the donation of molecular oxygen (6 and 5). As expected, biological process comprised the highest proportion following 12 and 19 days of culture: transmembrane transport (13 and 9), followed by carbohydrate metabolic process (2 and 2). While, the highest proportions of oxidoreductase activity involved in the donation of molecular oxygen (44 and 29), followed by metal ion binding (27 and 24), iron ion binding (22 and 16), heme binding (15 and 10), and N‐acetyltransferase activity (11 and 8) of the molecular function were highly upregulated in the submerged mycelia. In addition, transmembrane transport activity (40 and 22) was the highest proportion of biological process, followed by metabolic process (9 and 7), iron‐sulfur cluster assembly (7 and 6), fatty acid biosynthesis (7 and 5) and metal ion transport (3 and 3) that were upregulated. Overall, the findings indicated that substantial metabolic changes took place in the submerged mycelia compared to the aerial mycelia. In particular, the redox balance maintenance by the oxidoreductases and iron metabolism appears to be important in the submerged mycelia.

### 2.3 Pentose phosphate pathway and glycolysis

**Figure 5:**
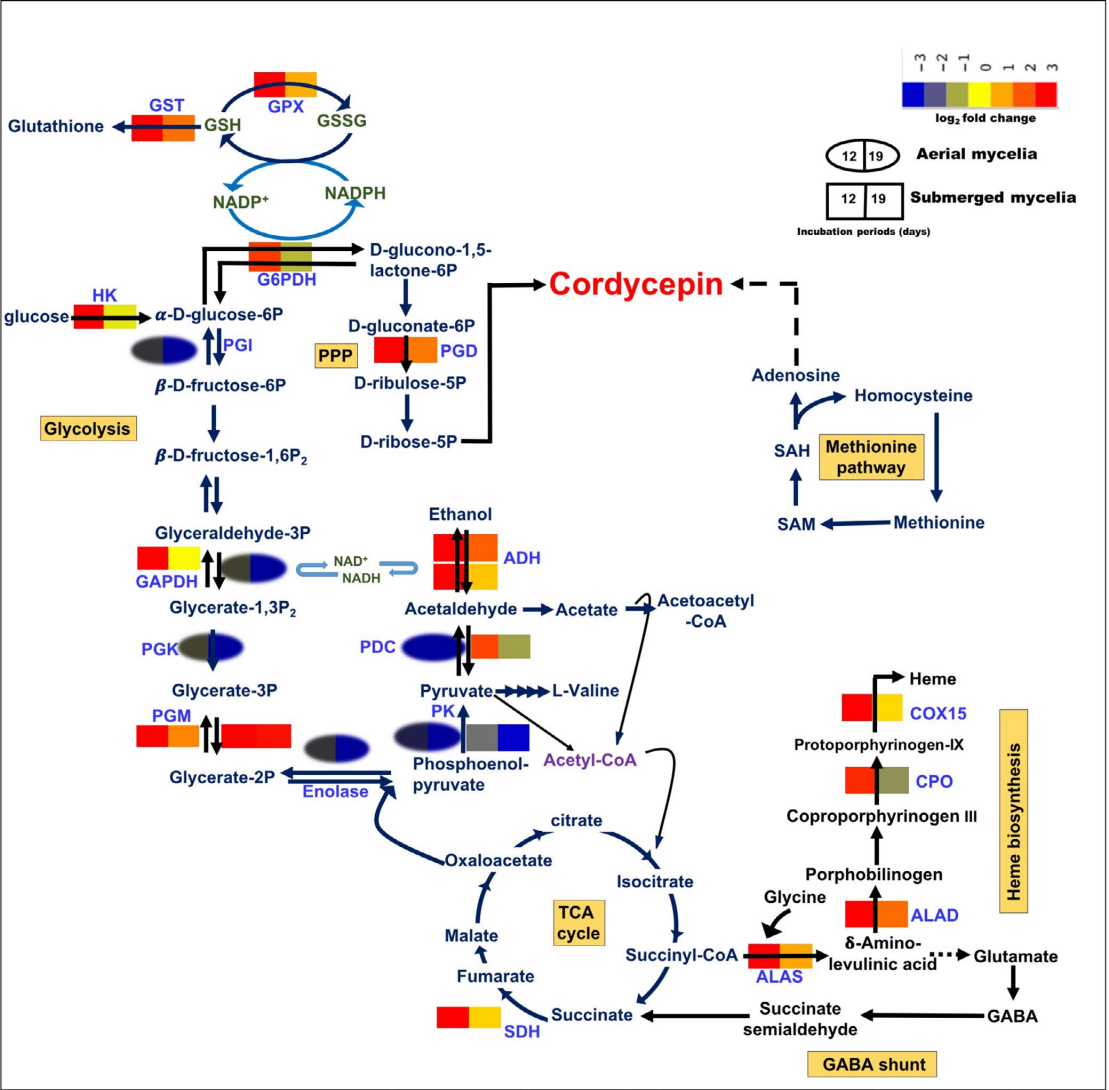
Metabolic pathway of aerial and submerged mycelia in the liquid surface culture of *Cordyceps militaris*. The expression of genes was significantly upregulated in the submerged mycelia and downregulated in the aerial mycelia. The glycolytic pathway was upregulated by the increasing activity of hexokinase (HK) to convert glucose to D‐glucose‐6P, followed by the activities of glyceraldehyde 3‐phosphate dehydrogenase (GAPDH) and phosphoglycerate mutase (PGM), which further enter the fermentation pathway, shown by the increasing activities of pyruvate decarboxylase (PDC) and alcohol dehydrogenase (ADH). Since the hypoxic condition was created in the submerged mycelia, the activities of some enzymes in the tricarboxylic acid (TCA) cycle were downregulated with the exception of the succinate dehydrogenase (SDH) enzyme. As a consequence, NADPH served as an energy carrier instead of ATP via the fermentation pathway and the activation of glucose‐6‐phosphate‐1‐dehydrogenase (G6PDH) and phosphogluconate dehydrogenase (PGD) enzymes of the pentose phosphate pathway (PPP). The PPP also plays a major role in providing the nucleotides for biosynthesis, as well as in cordycepin biosynthesis. Interestingly, under hypoxic conditions, this fungus also activated the heme biosynthetic pathway by upregulating the expression of 5‐aminolevulinic acid synthase (ALAS), delta‐aminolevulinic acid dehydratase (ALAD), coproporhyrinogen III oxidase (CPO), and cytochrome c oxidase (COX) enzymes, sequentially. However, the hypoxic condition is strongly related to the oxidative stress due to the production of reactive oxygen species (ROS). This fungus develops its defense mechanism by activating the expression of the peroxide family enzyme glutathione peroxidase (GPX) to reduce glutathione, which corresponds with the PPP biosynthesis using NADPH as a hydrogen donor and finally produces glutathione as an antioxidant using glutathione S‐transferase (GST) as a catalyst

The RNA‐Seq data showed that two genes in the pentose phosphate pathway (PPP), glucose‐6‐phosphate‐1‐dehydrogenase (G6PDH) (CCM_06983) and 6‐phosphogluconate dehydrogenase (PGD) (CCM_07716), were specifically upregulated in the submerged mycelia (Figure 5, Appendix Table A1). G6PDH and 6PGD produce NADPH in the cytosol. The activation of the PPP in hypoxic conditions has been observed in other fungi. In *Aspergillus nidulans*, the PPP was activated under hypoxic conditions to generate NADPH and produce pentose (Shimizu, Fujii, Masuo, Fujita, & Takaya, 2009).

In advance, the transcriptome results revealed that the Gal4 transcription factor that regulates the glycolysis metabolic pathway, CCM_09617, CCM_06477, CCM_03378, CCM_07868, CCM_06621, and CCM_08260*, *with fold numbers of expression of 6.065, 5.106, 4.107, 2.250, 2.518 and 2.955, was significantly upregulated in the submerged mycelia, and only CCM_08260 (4.182) was upregulated in the aerial mycelia. The expression of hexokinase (HK, CCM_06280), glyceraldehyde 3‐phosphate dehydrogenase (GAPDH, CCM_04549), and phosphoglycerate mutase (PGM, CCM_04218, CCM_09191) was also significantly upregulated following 12 days of culture in the submerged mycelia, and the expression level was reduced at 19th day (Figure 5). In addition, pyruvate kinase (CCM_06062) and phosphoglycerate kinase (CCM_08269) were downregulated in either the aerial or submerged mycelia during the culture periods (Appendix Table A1). Whereas, the glycolysis genes in *Trichoderma reesei* were upregulated under hypoxic conditions (Bonaccorsi et al., 2006).

### 2.4 Fermentation

Glycolysis is an essential type of metabolism to assimilate carbon via respiration or fermentation for entomopathogenic fungi without exception. Under hypoxic conditions, the physiology of the cell is adjusted by shifting the metabolism from oxidative phosphorylation and beginning to activate the fermentation pathway. In this study, the majority of the genes in the TCA cycle, such as pyruvate carboxylase, succinyl‐CoA synthetase and malate dehydrogenase, was repressed in the aerial mycelia (Figure 5).

**Figure A1:**
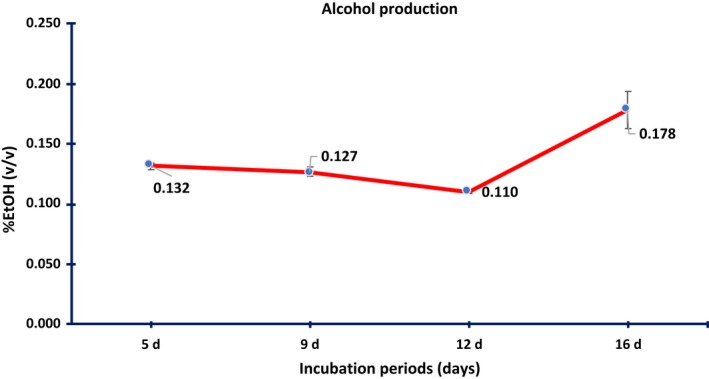
Ethanol assay of the liquid‐surfaced culture medium of Cordyceps militaris. *Error bars represent standard deviation (*SD*) of triplicate samples

In the submerged mycelia, the expression of the gene encoding pyruvate decarboxylase (PDC, CCM_01231) was upregulated at 12th day but downregulated in its aerial mycelia on the same culture time. Alcohol dehydrogenase 1 (ADH, CCM_02484) displayed the same pattern of expression and was upregulated in the submerged mycelia at 12th day but not in the aerial mycelia (Figure 5). Six putative ADHs (CCM_01806, CCM_09633, CCM_00716, CCM_08262, CCM_02861, and CCM_03437) were upregulated, while three others (CCM_09031, CCM_00356, and CCM_09512) were significantly downregulated (Figure 5). However, ethanol production was not detected in this study (Appendix Figure A1), and most of these genes may encode medium‐chain dehydrogenase (MDR) family proteins instead of ADHs. L‐lactate dehydrogenase (CCM_08025) was downregulated in both types of mycelia throughout the cultivation periods (Appendix Table A1). However, ethanol production was observed in hypoxic conditions in *Aspergillus *sp. (Grahl et al., 2011; Masuo et al., 2010).

### 2.5 Heme and siderophore biosynthesis

Heme biosynthesis is composed of eight enzymes, including 5‐aminolevulinic acid synthase (ALAS), delta‐aminolevulinic acid dehydratase (ALAD), porphobilinogen deaminase (PBGD), uroporphyrinogen III synthase (UROS), coproporphyrinogen III oxidase (CPO), protoporphyrinogen oxidase (PPO), cytochrome c oxidase (COX), and ferrochelatase (FC) (Franken et al., 2011). Interestingly, four of the heme biosynthetic genes, ALAS (CCM_01504), ALAD (CCM_00935), CPO (CCM_07483), and COX 15 (CCM_05057) with 3.176‐, 4.660‐, 4.239‐, and 2.526‐fold expression, were activated only in the submerged mycelia following 12 days of culture (Figure 5). Nevertheless, the expression of CPO was downregulated at 19th day. Interestingly, the expression level of ALAD was the highest among the others and consistent with a previous study of ALAD, which is hypothesized to play a role as the rate limiting step of heme biosynthesis in *N. crassa *(Chandrika & Padmanaban, 1980; Gibson, Havens, Metz, & Hilf, 2001). The putative flavohemoprotein (CCM_5119), which requires heme for its activity, was specifically expressed in the submerged mycelia following 12 days of culture.

In addition, the expression of the siderophore iron transporters mirB (CCM_08808), mirC (CCM_01166), FtrA (CCM_05132), sit1 (CCM_08222), and Atx1, putative (CCM_02485) and the iron‐related transporters (CCM_02485, CCM_08222, CCM_05132, CCM_07591, and CCM_01166) involved in the metabolism of iron were differentially expressed at 12th day in the submerged mycelia. Indeed, the iron ion transporters mirB (CCM_07826) and mirC (CCM_01166) were upregulated and downregulated, respectively, at 12th and 19th day. The mechanism of the secretion of the siderophores strongly corresponds to the efflux pump system of the major facilitator superfamily and the ATP‐binding cassette (ABC) superfamily transporter (Miethke, Schmidt, & Marahiel, 2008; Nicolaisen et al., 2010; Rodriguez & Smith, 2006). In some fungi, hypoxia activates heme biosynthesis, iron uptake and iron metabolism as found in this RNA‐Seq data, which are consistent with previous results (Blatzer et al., 2011; Chang, Bien, Lee, Espenshade, & Kwon‐Chung, 2007).

The most striking observation to emerge from the data comparison was that the genes of the ABC multidrug transporter and drug resistance were highly expressed in the submerged mycelia, including 12 genes consisting of five ABC transporter genes (CCM_06618; CCM_01696; and CCM_08836, which are nucleotide‐binding domain features; CCM_04694 and CCM_01393), and seven genes involved in multidrug resistance (CCM_00309; CCM_02386; CCM_06620; CCM_04242; CCM_08649; CCM_01312; and CCM_00623). In contrast, only the two genes CCM_07735 and CCM_00608 were found in the aerial mycelia. Moreover, the activities of the four genes encoding the iron‐sulfur cluster proteins (CCM_01611, CCM_02863, CCM_06154, and CCM_07146) were also only found in the submerged mycelia (Appendix Table A1).

### 2.6 Reactive oxygen species and the antioxidant defense system

**Figure 6:**
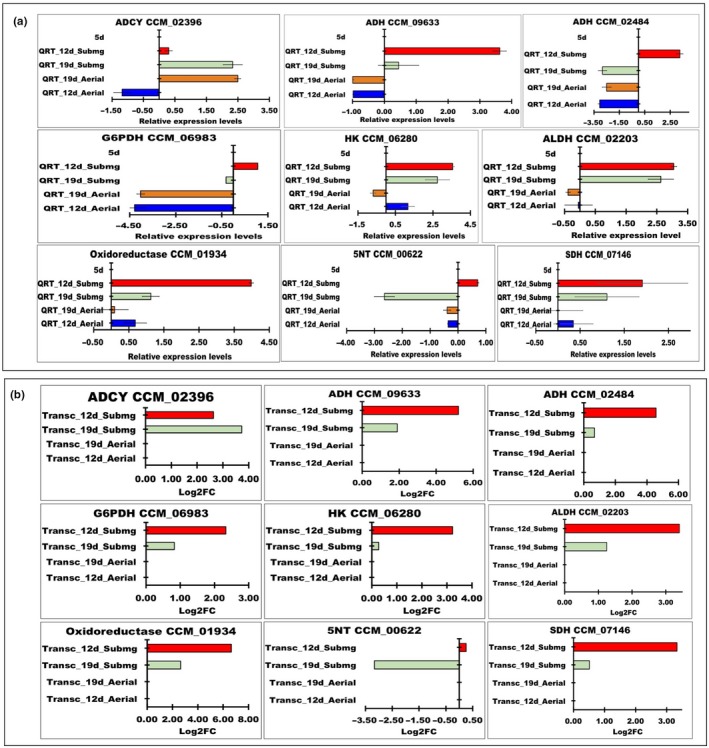
(a) Quantitative RT‐PCR of the DEGs. The highest quantitative result was shown by oxidoreductase (CCM_01934), followed by alcohol dehydrogenase (ADH; CCM_09633), aldehyde dehydrogenase (ALDH; CCM_02203), hexokinase (HK; CCM_06280), alcohol dehydrogenase (ADH; CCM_02484), succinate dehydrogenase (SDH; CCM_07146), glucose‐6‐phosphate‐1‐dehydrogenase (G6PDH; CCM_06983), 5′‐nucleotidase (5NT; CCM_00622), and adenylate cyclase, putative (ADCY; CCM_02396) in the submerged mycelia. In contrast, in the aerial mycelia, only adenylate cyclase, putative (ADCY; CCM_02396) and oxidoreductase (CCM_01934) showed the highest levels of expression, while the other enzymes were downregulated. (b) Transcriptomic analysis results

In some fungi, the connection of hypoxia with oxidative stress and the production of reactive oxygen species (ROS) is hypothesized (Grahl, Shepardson, Chung, & Cramer, 2012; Hillmann, Shekhova, & Kniemeyer, 2015). This RNA‐Seq study showed that the cytosolic Cu/Zn superoxide dismutase (SOD) (CCM_07115), which catalyzes the dismutation of the superoxide anion radical to oxygen and H_2_O_2_, was found at 12th day in the submerged mycelia of *C. militaris*. The second barrier mechanism of defense against H_2_O_2_ was subsequently activated by increasing the activity of the peroxidase class glutathione peroxidase family protein (GPX, CCM_03086) and cytochrome c peroxidase (CCM_06954). Glutathione peroxidase reduces hydrogen peroxide using NADPH as a reductant (Figure 6). The superoxide anion radical and hydrogen peroxide were converted to H_2_O by this SOD and GPX (Breitenbach, Weber, Rinnerthaler, Karl, & Breitenbach‐Koller, 2015; Guevara‐Flores, Martínez‐González, Rendón, & Arenal, 2017). A fivefold expression level of glutathione S‐transferase GstA (GST, CCM_06544) and a threefold expression level of the glutamate‐cysteine ligase catalytic subunit (CCM_00539), putative microsomal glutathione S‐transferase 3 (CCM_03961), and ribonucleoside‐diphosphate reductase M2 subunit (CCM_05761) were found in the submerged mycelia. However, these genes were not expressed in the aerial mycelia.

In this study, the expression of two peroxiredoxins, putative peroxiredoxin‐5 (CCM_03275), peroxiredoxin Osmc‐like protein (CCM_06109), four thioredoxins (thioredoxin‐like protein (CCM_03715), putative cytoplasmic thioredoxin (CCM_00029), thioredoxin (CCM_00331), M‐type thioredoxin (CCM_02074) and thioredoxin reductase (CCM_05420) was found as upregulated proteins in the submerged mycelia following 12 days of culture (Appendix Table A1, Supplementary Table S1). Organic peroxides are converted to alcohols by peroxiredoxins, which are conjugated with thioredoxins and thioredoxin reductase (Breitenbach et al., 2015).

### 2.7 Validation of RNA‐Seq using quantitative RT‐PCR

The consistency of the RNA‐Seq experiments was confirmed using qRT‐PCR. Nine DEGs from the glycolysis pathway, fermentation and putative cordycepin biosynthetic pathway were selected, and primers were designed for qRT‐PCR. The quantification results showed that oxidoreductase (CCM_01934) was the most strongly expressed, followed by alcohol dehydrogenase (ADH, CCM_09633), aldehyde dehydrogenase (ALDH; CCM_02203), hexokinase (HK; CCM_06280), alcohol dehydrogenase (ADH; CCM_02484), succinate dehydrogenase (SDH; CCM_07146), glucose‐6‐phosphate‐1‐dehydrogenase (G6PDH; CCM_06983), 5′‐nucleotidase (5NT; CCM_00622), and adenylate cyclase, putative (ADCY; CCM_02396) at 12th day and maintained in the 19 days culture of the submerged mycelia (Figure 6). In the aerial mycelia, the activities of ADH, SDH, G6PD and HK were upregulated, while the other genes were downregulated. In addition, only ADCY, putative (ADCY; CCM_02396) and oxidoreductase (CCM_01934) were highly expressed in the aerial mycelia, while the other enzymes were downregulated, especially 5‐NT on 12th day (Figure 6). Overall, the expression profiles of qRT‐PCR were similar to the transcriptome results.

### 2.8 Metabolites in the culture medium during the liquid surface culture

**Figure 7:**
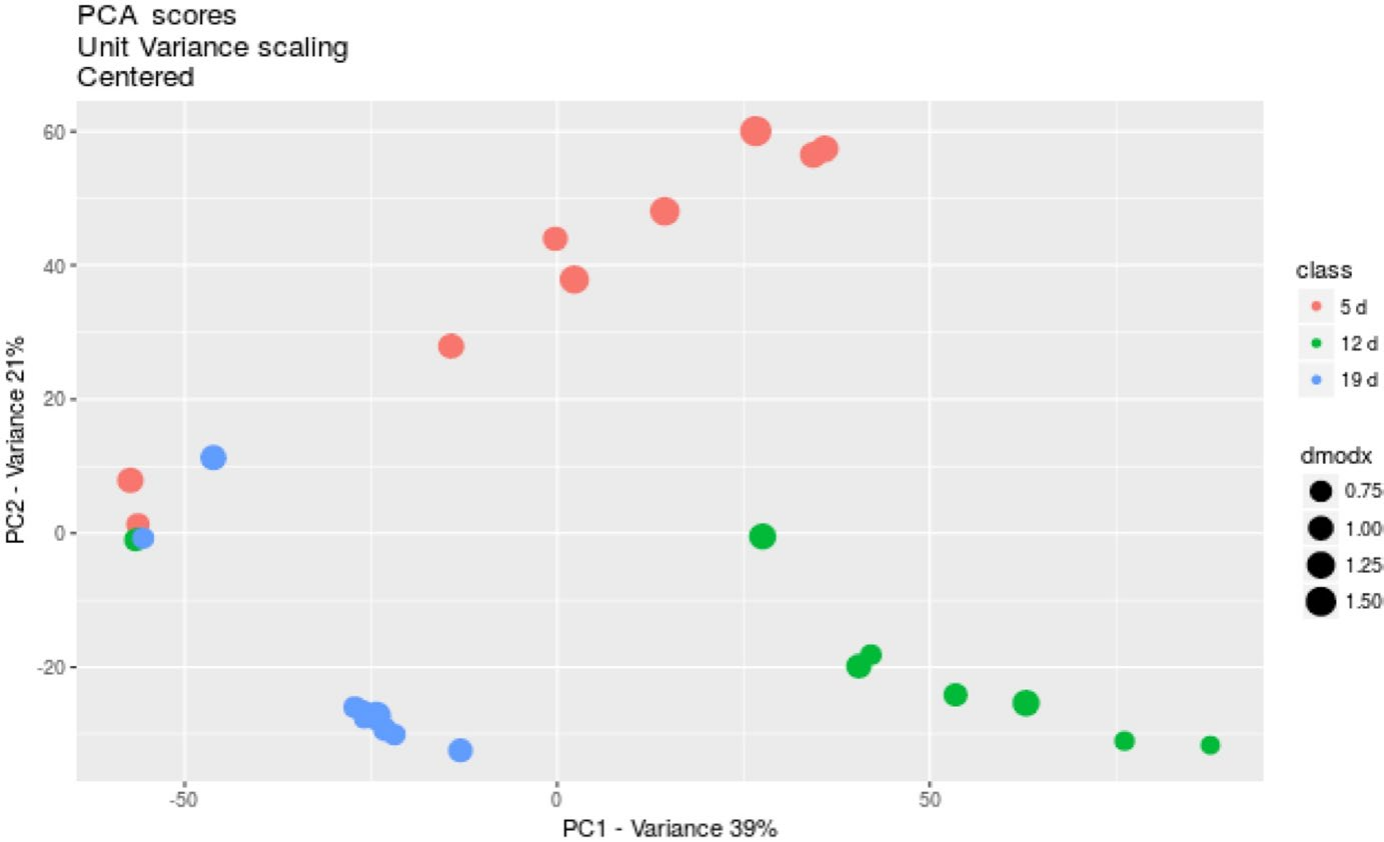
PCA score of the different clusters of the sampling days and the predictive annotation of the metabolites of the liquid surface culture. PCA score plots of the metabolites from each sample showed that the variation value between the samples in the PC1 group was higher than the variation value within the samples in the PC2 group, with counts value of 39% and 21%, respectively. The predictive metabolites of valine were detected throughout the cultivation periods. Alanine, glycine, inositol, and urea were detected at either 12th day or 19th day. Interestingly, adenosine was only detected at 12th day

In advance, to investigate the metabolites during the liquid surface culture, GC‐MS analysis was conducted. Figure 7 shows the principal component analysis (PCA) score plots of the metabolites from each sample. As expected, the plot shows that the variation value between the samples group (PC1) was higher than the variation within the samples group (PC2), with count values of 39% and 21%, respectively. This indicated that the metabolism following 5, 12, and 19 days of culture was different. As expected, some amino acid metabolites were detected in the media, such as guanine, L‐arginine, L‐ ornithine, adenine and xanthosine, a metabolite product of purine metabolism were leveled up at 12th day of the culture periods but not at 19th day. Consistent with the transcriptomic result, the heme metabolites coproporphyrinogen III and bilirubin were enriched and declined at 12th day and 19th day, respectively. In contrast, (S)‐malate a metabolite product of TCA and glycine metabolite of glutathione‐mediated detoxification were solely found following 5 days of culture. While, adenine level derived from adenine and adenosine salvage III pathway was enriched on 19th day (Table 1). Glycine is a precursor of heme, and its production corresponds to the activation of the heme biosynthetic pathway under hypoxic conditions. The 3‐hydroxy‐L‐kyurenine and indole‐3‐ethanol metabolite products of tryptophan degradation were significantly declined in the submerged mycelia at 19th day. However, cordycepin was not detected using this GC‐MS protocol. The consumption of phenylalanine suggested that it could be a candidate of additives to enhance the production of cordycepin. However, in our previous study, the addition of phenylalanine did not have any effect on cordycepin production in *C. militaris* (Sari, Suparmin, Kato, & Park, 2016).

**Table 1:** Predictive metabolites in liquid surface culture

Metabolite	Pathway	Up/ Down	Fold Change	*p*‐value	*m/z*	RT
Metabolites Prediction 5 d VS 12 d						
4‐fumaryl‐acetoacetate	Tyrosine metabolism	Up	45.3	0.0047	201.0392	8.10
(S)‐malate	TCA cycle	Down	16.10	0.0003	68.0192	9.57
Xanthosine	Purine ribonucleosides degradation to ribose‐1‐phosphate	Up	4.50	0.0045	154.0351	9.92
2‐(formamido)‐N1‐(5‐phospho‐β‐D‐ribosyl) acetamidine	5‐aminoimidazole ribonucleotide biosynthesis	Up	3.80	0.0046	169.0347	11.40
L‐arginino‐succinate	Urea cycle	Down	59.40	0.0002	293.1479	293.1479
Guanine	Purine ribonucleosides degradation to ribose‐1‐phosphate	Up	3.00	0.0001	169.0808	12.35
L‐arginine	urea cycle	Up	4.00	0.0040	158.0915	12.67
(R)‐mevalonate	Mevalonate pathway	Up	5.20	0.0017	171.0612	12.86
Coproporphyrinogen III	Heme biosynthesis from uroporphyrinogen‐III	Up	2.70	0.0047	342.1556	14.73
L‐ornithine	Glutathione metabolism	Up	2.60	0.0029	173.0680	15.01
L‐tryptophan	Glycine, serine and threonine metabolism	Up	2.40	0.0009	189.0789	16.34
(S)‐2‐amino‐6‐oxohexanoate	lysine degradation II (pipecolate pathway)	Down	2.90	0.0018	169.0714	16.95
Delta‐1‐piperideine 6‐carboxylate	lysine degradation II (pipecolate pathway)	Down	2.10	0.0011	110.0610	17.04
Glycine	Glutathione‐mediated detoxification	Down	3.10	0.0014	99.0302	17.52
L‐lysine	Lysine degradation I (saccharopine pathway)	Down	2.90	0.0000	187.0832	17.53
Metabolites Prediction 5d VS 19d						
4‐methyl‐2‐oxopentanoate	Valine, leucine and isoleucine degradation	Up	15.00	0.00047	114.0421	6.32
(R)‐propane‐1,2‐diol	methylglyoxal degradation VI	Up	36.40	0.000052	60.0333	7.82
2‐aminoprop‐2‐enoate	L‐serine degradation	Down	261.90	0.00012	88.0386	9.49
L‐cysteine	Cysteine biosynthesis/homocysteine degradation (trans‐sulfuration)	Down	12.20	0.000017	105.0227	9.56
Adenine	Purine metabolism	Down	30.00	0.000093	119.0364	9.57
L‐glutamate	Alanine, aspartate and glutamate metabolism	Up	6.40	0.0003	149.0671	10.39
L‐serine	Glycine, serine and threonine metabolism	Down	9.30	0.00078	129.0406	10.63
L‐serine	glutathione‐mediated detoxification	Up	6.60	0.00048	155.0503	11.51
L‐threonine	Glycine, serine and threonine metabolism	Down	3.50	0.00069	103.0609	11.76
(R)‐pantothenate	Coenzyme A biosynthesis	Down	7.40	0.0001	220.1138	11.88
L‐ornithine	Ornithine de novo biosynthesis	Down	3.80	0.00031	173.0679	12.18
Bilirubin	Heme degradation	Down	10.70	0.00033	293.1422	12.4
L‐histidine	Histidine metabolism	Down	4.70	0.00084	140.0575	14.08
L‐phenylalanine	Phenylalanine degradation/tyrosine biosynthesis	Up	5.00	0.000061	189.0796	14.85
Indole‐3‐acetate	tryptophan degradation via tryptamine	Down	7.20	0.0003	193.0936	16.99
3‐hydroxy‐L‐kynurenine	tryptophan degradation to 2‐amino‐3‐carboxymuconate semialdehyde	Down	6.00	0.0000	243.1244	17.03
(S)‐dihydroorotate	UMP biosynthesis	Down	6.10	0.0001	176.0644	17.54
Glycine	Glycine/serine biosynthesis	Down	5.30	0.0006	99.0302	17.55
Glutathione	Glutathione biosynthesis	Down	4.30	0.0001	155.056	17.56
3‐methyl‐2‐oxobutanoate	Valine degradation	Down	6.50	0.0002	141.0752	17.57
Dimethylglycine	Glycine betaine degradation	Down	5.00	0.0001	127.0603	17.59
S‐adenosyl 3‐(methylthio)propylamine	Spermidine biosynthesis	Down	3.60	0.0008	179.0871	17.61
S‐methyl‐L‐methionine	Methionine salvage	Down	5.60	0.0002	149.0636	17.62
2'‐deoxyuridine	Pyrimidine deoxyribonucleosides salvage	Down	4.70	0.0000	246.1128	17.63
β‐alanine	Pyrimidine metabolism	Down	6.70	0.0002	113.0455	17.66
Coproporphyrinogen III	Heme biosynthesis from uroporphyrinogen‐III	Down	8.50	0.0001	331.1646	17.68
biliverdin‐IX‐α	Heme degradation	Down	5.30	0.0000	292.1355	18.28
Metabolites Prediction 12 d VS 19 d						
L‐2‐aminoadipate	Lysine degradation I (saccharopine pathway)	Down	41.50	0.0043	201.0392	8.09
L‐histidine	Histamine biosynthesis	Down	4.90	0.000	157.0827	9.86
Xanthosine	Purine ribonucleosides degradation to ribose‐1‐phosphate	Down	4.90	0.0027	154.0346	9.89
indole‐3‐ethanol	Tryptophan degradation via tryptamine	Down	81.60	0.0011	92.5396	10.06
L‐cystathionine	Cysteine biosynthesis/homocysteine degradation (trans‐sulfuration)	Down	5.50	0.0036	124.0381	10.38
Pyridoxine	Pyridoxal 5'‐phosphate salvage	Down	15.80	0.0014	170.0784	10.59
L‐serine	Glycine, serine and threonine metabolism	Down	8.50	0.0002	129.0402	10.63
choline	Choline degradation	Down	56.00	0.0000	87.1028	10.78
5‐methoxytryptamine	Tryptophan metabolism	Up	5.60	0.0025	229.0747	11.23
2'‐deoxyuridine	Pyrimidine Deoxyribonucleosides degradation	Up	23.30	0.0011	113.0288	11.48
3‐hydroxyanthranilate	tryptophan degradation to 2‐amino‐3‐carboxymuconate semialdehyde	Up	11.50	0.0006	171.0792	11.51
(R)‐propane‐1,2‐diol	Methylglyoxal degradation VI	Up	22.40	0.0011	99.0419	11.54
L‐glutamate	Alanine, aspartate and glutamate metabolism	Up	5.10	0.0024	132.0395	11.70
3‐phospho‐hydroxypyruvate	Glycine, serine and threonine metabolism	Up	3.20	0.0050	166.9733	11.89
Adenine	Adenine and adenosine salvage III	Up	3.00	0.0020	180.0507	12.03
2‐oxobutanoate	Cysteine biosynthesis/homocysteine degradation (trans‐sulfuration)	Up	2.20	0.0003	120.0675	12.13
S‐methyl‐L‐methionine	methionine salvage	Down	17.40	0.0002	146.0665	14.27
S‐adenosyl 3‐(methylthio)propylamine	Spermidine biosynthesis	Down	4.50	0.0042	374.1916	17.80
3‐hydroxy‐L‐kynurenine	Tryptophan degradation to 2‐amino‐3‐carboxymuconate	Down	4.40	0.0029	226.0924	19.81

## 3 DISCUSSION

### 3.1 Global metabolism changes in submerged mycelia

The submerged mycelia of *C. militaris *activated metabolic shifting to control the homeostasis of intracellular redox under hypoxic conditions. These results corroborate the findings of a substantial amount of previous studies on cell adaptation in hypoxic conditions by activated the metabolic pathways of iron, heme biosynthesis, glycolysis, the PPP, and fermentation (Grahl et al., 2012; Takaya, 2009). It is possible that the upregulation of some alcohol dehydrogenases might be related to the metabolism of alcohol as a product of detoxification by peroxidase, which also resulted in NAD(P)H rather than the fermentation pathway. Moreover, some studies suggested that secondary metabolites are synthesized by submerged mycelia (Granozzi, Billetta, Passantino, Sollazzo, & Puglia, 1990; Novotna et al., 2003; Papagiani, 2004).

We recently reported that cordycepin achieved the highest production in the 15 days of the liquid surface culture of *C. militaris* and hypothesis that cordycepin biosynthesis coincides with hypoxia (Suparmin et al., 2017). The primary objective of this study was to clarify the understanding of cordycepin production in the liquid surface culture, examine the hypoxic condition in detail and determine whether the aerial or submerged mycelia contributed to cordycepin biosynthesis. Hypoxia produces superoxide (ROS), and the cells respond to it by activating SOD (CCM_07115) followed by glutathione peroxidase (CCM_03086) and cytochrome c peroxidase (CCM_06954) with 5.157‐ and 5.570‐fold expression, respectively. This indicates that the submerged mycelia more actively reduced the peroxide than the aerial mycelia using NADPH as a reductant. As reported that the addition of ferrous sulfate could enhance the cordycepin production and the activity of superoxide dismutase (SOD) related to the cordycepin production in the fruiting body of *C. militaris *(Dong, Lei, Ai, & Wang, 2012; Fan, Wang, & Zhong, 2012). The formation of ROS also elaborated the stabilization of hypoxia‐inducible factors (HIF) under hypoxic conditions using proline hydroxylase as a catalyst (Chandel et al., 2000; McGovern et al., 2011; Schroedl, McClintock, Budinger, & Chandel, 2002; Semenza, 2002). Superoxide can also be produced through nonenzymatic mechanisms that utilize coenzymes or prosthetic groups, flavins or the iron‐sulfur cluster.

The biosynthesis of the iron‐sulfur clusters in eukaryotes and bacteria requires NADPH as a cofactor. To maintain NADPH production, the cells increased the activities of G6PDH in the pentose phosphate pathway (PPP) (Giro, Carrillo, & Krapp, 2006). The PPP plays a major role in providing the precursors of nucleotide biosynthesis, as well as producing NADPH and thus maintaining homeostasis (Stincone et al., 2015; Tarrío, García‐Leiro, Cerdán, & González‐Siso, 2008). In addition, a substrate of cordycepin biosynthesis might be provided from the methionine pathway even though the expression of the genes was not significantly different (Appendix Table A1). NADPH oxidase has two hemes as a cofactor to catalyze the production of superoxide anion radicals by the reduction of oxygen with NADPH (Lamberth, 2004). Iron, which is sequestered in the form of heme, is an essential cofactor for activating some pathogenesis genes during host infection. In this study, heme biosynthesis and iron uptake were significantly upregulated following 12 days of culture.

Iron acquisition and transportation via siderophores are a common strategy in pathogenic fungi to control iron homeostasis in cells that are also mediated by SreA. A homologous siderophore component Mdr1 in *Aspergillus fumigatus* was annotated as ABC multidrug transporter Mdr1 (CCM_02386) and was significantly upregulated in the submerged mycelia with 4.776‐fold expression at 12th day and maintained to 1.640‐fold expression at 19th day. The overexpression of Mdr1 and AtrF from *A. fumigatus* reduced the sensitivity to echinocandin and itraconazole, respectively (Slaven et al., 2002; Tobin, Peery, & Skatrud, 1997). Therefore, it can be hypothesized that *C. militaris *developed defense mechanisms by activating some multidrug resistance genes, such as CCM_00309, CCM_02386; CCM_06620, CCM_08649 and CCM_04242 and included ABC transporters to decrease the oxidative stress.

### 3.2 Heme biosynthesis in submerged mycelia

It is worth noting that the normal biosynthesis of heme requires oxygen as a substrate of coproporphyrinogen and protoporphyrinogen oxidase. However, these results were in contrast to the best‐known fungal model *Saccharomyces cerevisiae *in which heme biosynthesis is reduced under hypoxic conditions (Franken et al., 2011). This study supports evidence from previous observations of the array analysis of heme biosynthetic genes in the other fungi including *S. pombe*, *Cryptococcus neoformans*, and *A. fumigatus, *which are induced during hypoxia (Blatzer et al., 2011; Chang et al., 2007; Hughes, Todd, & Espenshade, 2005).

Among the differentially upregulated genes in heme biosynthesis, ALAD (CCM_00935) had the highest expression fold increase and the coproporphyrinogen III metabolite was confirmed in the medium at 12th day culture periods. Thus, it can be assumed that ALAD might have a dual regulatory function in this fungus. In addition, CCM_07483 encodes a protein with high similarity to Hem13 of the CPO from *A. fumigatus *with 53% identity and 98% homology that was also upregulated. This Hem13 is controlled by the GATA factor sterol regulatory binding protein SreA. SreA is also known as a hypoxia response transcriptional regulator (Chung, Haas, & Cramer, 2012; Schrettl et al., 2008). A homolog to the zinc finger transcription factor Upc2 from *Candida albicans, *the C6 transcription factor Zn (2)‐Cys (6) (CCM_07141) that regulates SreA was also found (MacPherson et al., 2005; Synnott, Guida, Mulhern‐Haughey, Higgins, & Butler, 2010). These genes are strongly associated with glycolysis, oxidative stress resistance, cell wall biosynthesis, ergosterol biosynthesis and iron acquisition (Blatzer et al., 2011; Willger et al., 2008). As expected, upregulated expression of the SRE1 homologs in the ergosterol biosynthetic pathway, such as CCM_07184, CCM_07586, CCM_07840, CCM_01839, and CCM_02962, was found in the submerged mycelia.

**Figure 8:**
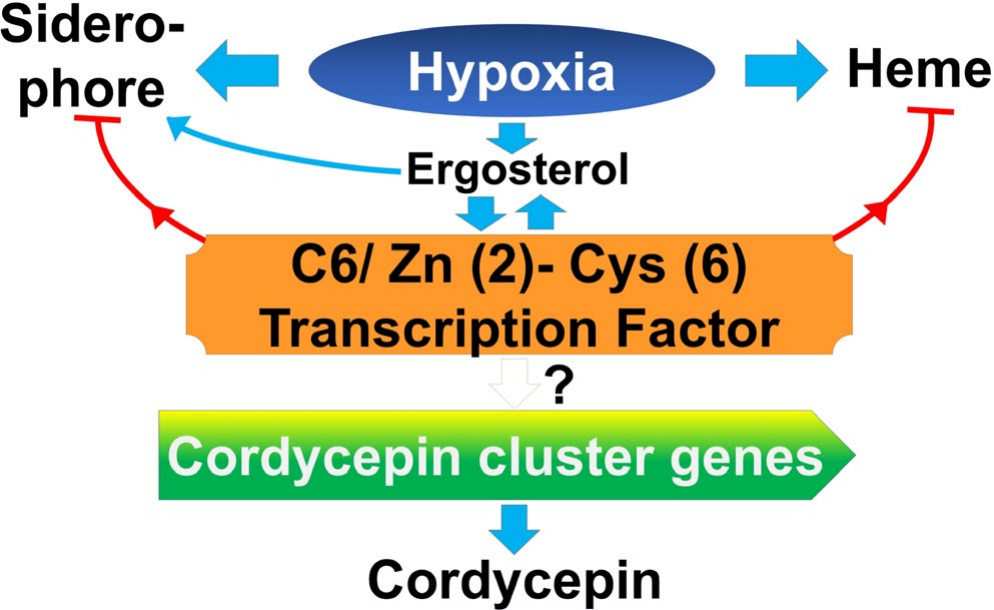
Proposed correlation between hypoxia and the regulation of cordycepin biosynthesis in liquid surface culture of *Cordyceps militaris. *Hypoxic conditions induced the activation of siderophores to take up the iron for heme biosynthesis. Simultaneously, the conditions also activated the ergosterol biosynthesis, which is also induced by siderophores. The C6 transcription factor Zn (2)‐Cys (6) (CCM_07141) regulated the ergosterol biosynthesis and heme biosynthesis and the iron cluster genes of cordycepin biosynthesis that might be partially regulated by the C6 transcription factors (TFs) (CCM_07141). Finally, the presence of cordycepin suggested the inhibition of the C6 TFs and the cluster genes of cordycepin biosynthesis

Taken together, this combination of results may explain the correlation of hypoxia and cordycepin production in the liquid surface culture. Also, their mechanism of protection of submerged mycelia is by producing the ergosterol. Even though, the expression of the cluster genes of cordycepin biosynthesis was not significantly different in both types of mycelia. It indicated that the whole mycelia are involved in cordycepin biosynthesis (Figure 8). These studies help to elucidate the contribution of the submerged mycelia in the pathogen‐host interaction and their pathogenesis and are an important issue for future research and the in vivo role of cordycepin in *C. militaris*.

## 4 MATERIALS AND METHODS

### 4.1 Strain, media and culture conditions

*C. militaris *strain NBRC 103,752 was purchased from the Biological Research Center (NITE, Tokyo, Japan) and used throughout this research. The mycelia of the strain were dissolved in 1 ml of medium, which was composed of 5 g/L peptone, 3 g/L yeast extract, and 1 g/L MgSO_4_.7H_2_O. The liquid medium was transferred to potato dextrose agar (PDA) (Nissui Pharmaceutical Co. Ltd., Fuji, Japan) and incubated for 7 days at 25°C. Mycelia in the PDA plates were used for cultivation in the liquid media, and the PDA slants were stored at 4°C as a stock culture.

This fungus was inoculated into an optimized culture medium (Sari et al., 2016) composed of 72.5 g/L yeast extract, 62.6 g/L glucose (pH 5.6) and Vogel's medium with 1/10 concentration containing 0.28 g/L sodium citrate dihydrate, 0.50 g/L KH_2_PO_4_, 0.20 g/L NH_4_NO_3_, 0.02 g/L MgSO_4_·H_2_O, 0.01 g/L CaCl_2 _2H_2_O, 0.46 × 10^−3^ g/L citric acid, 0.50 × 10^−3^ g/L ZnSO_4_, 0.1 × 10^−3^ g/L Fe(NH)_4_(SO_4_)_2_.6H_2_O, and 0.025 × 10^−3^ g/L CuSO_4_ 5H_2_O and incubated at 25°C for 19 days in liquid surface culture.

### 4.2 Analytical methods

The samples were thawed and centrifuged at 15,000× *g* at 4°C for 10 min. The supernatant was mixed with 2% methanol at a 1:1 ratio and filtered through a 0.45 μm filter (Cat No. HAWP04700; Millipore, Billerica, MA) before analysis. The cordycepin concentration in the supernatant was measured using high performance liquid chromatography with a UV detector at 260 nm (Shimadzu, Tokyo, Japan). The TSK‐gel ODS‐80Ts (Tosoh Corp., Tokyo, Japan) was used at 40°C with 0.1% phosphoric acid: methanol at a 98:2 ratio (v/v) as the mobile phase (Masuda, Das, Hatashita, Fujihara, & Sakurai, 2014). Cordycepin (Wako Pure Chem. Ind. Ltd., Osaka, Japan) was used as the reference standard.

### 4.3 Raw data processing, de novo assembly and differential gene expression analysis

*C. militaris *mycelia grown in the liquid surface culture for 5, 12 and 19 days were used for RNA extraction, sequentially. The RNA was extracted from both the aerial and submerged mycelia using TRIzol reagent (Invitrogen, Carlsbad, CA) according to the manufacturer's instructions. Briefly, 100 mg of mycelia was added to a 2 ml Eppendorf tube, homogenized in liquid nitrogen, and 1 ml of TRIzol was directly added. The mixture was incubated for 5 min at room temperature, and 200 μL of chloroform in 1 ml of TRIzol was added followed by centrifugation at 12,000× *g* for 15 min. The supernatant was removed, and the resulting pellet was washed with 1 ml of 75% ethanol. The pellet was dissolved in 50 μL of nuclease‐free water. The total RNA was treated using RNase‐free DNase I (Qiagen, South San Francisco, Canada). At 12th and 19th day, total RNA was extracted separately from the aerial and submerged mycelia. However, total RNA was extracted from the whole mycelia at 5th days because the aerial and submerged mycelia were not sampled separately.

RNA‐Seq was conducted by Eurofins Genomic K.K. (Tokyo, Japan). In brief, total RNA samples (800 ng) extracted from the submerged mycelia and aerial mycelia (*n* = 2 each) were used for strand‐specific RNA‐Seq library construction using an Illumina HiSeq2500 with the sequence mode 2 × 125 bp. The reads were cleaned by Trimmomatic (Ver.0.32) (Bolger, Lohse, & Usadel, 2014) to remove adaptor sequences and low‐quality reads and mapped to the *C. militaris *RNA assembly (ftp://ftp.ncbi.nlm.nih.gov/genomes/all/GCF/000/225/605/) from NCBI using BWA (Ver. 0.7.10) (Li et al., 2009).

Differentially expressed genes among the samples prepared at 5th, 12th and 19th day were identified using edgeR (Ver.3.16.1) (Robinson, McCarthy, & Smyth, 2010) with Trimmed Mean of M‐values (TMM) normalization methods (Robinson & Oshlack, 2010) to normalize for the RNA composition by finding a set of scaling factors for the library sizes. Upregulated and downregulated genes were defined with a log 2‐fold change (log FC) ≥1 and ≤1, respectively, with a false discovery rate (FDR) cutoff of 5%.

### 4.4 Gene ontology (GO) enrichment analysis

DEGs of the submerged and aerial mycelia were independently analyzed for enriched Gene ontology (GO), and the KEGG pathway was analyzed using DAVID software 6.8 (https://david.ncifcrf.gov/tools.jsp) (Huang, Sherman, & Lempicki, 2009).

### 4.5 GC/MS analysis

Fifty milliliters of *C. militaris *liquid surface culture broth at 5th, 12th, and 19th day was separately collected and stored at −80°C before further analysis. Each sample of 15 ml was diluted in 35 ml of water, centrifuged at 16,000× *g* for 10 min at 4°C, and the pellets were collected for freeze‐drying. Twenty‐five microliters of methoxyamine hydrochloride in pyridine (20 mg/ml) was added, and the mixture was vortexed for 30 s and incubated at 90°C for 90 min. Finally, 75 μL of N‐methyl‐N‐trimethyl‐silyltrifluoroacetamide (MSTFA) was added, and the mixture was incubated at 37°C for 30 min. The mixture was centrifuged at 13,000× *g* for 10 min at 4°C, and the supernatant (*n* = 7 for each sample) was subjected to gas chromatography/mass spectrometry (GC/MS) (Agilent Technologies 7890A GC system, Agilent Technologies, Inc., Wilmington, DE) equipped with an inertcap 5MS capillary column (5% phenylmethylsiloxane: 30 m × 0.25 mm internal diameter, 0.25 μm film thickness; GL‐science Co. Ltd., Tokyo, Japan) and JEOL JMS‐T100GCv Time‐of‐flight Mass Spectrometer (JEOL, Tokyo, Japan). The GC was operated at a constant flow of helium (1 ml/min), an injector temperature of 250°C, and an ion source and transfer line temperature of 280°C. The oven temperature program was as follows: 40°C for 4 min, increased at 15°C/min to 300°C, and held for 10 min. The samples were injected with a split ratio of 100:1. The ionization was conducted in the EI positive mode. The detection mass range was m/z 50–600. To tentatively identify a compound, the mass spectra and measured exact mass were compared against a spectral library (NIST) and the exact mass simulated. The spectra, exact mass, and retention times were compared with authentic standards when they were available.

### 4.6 Data statistics

The GC‐MS raw results were converted to. *cdf* format and analyzed using an XCMS online program (https://xcmsonline.scripps.edu/landing_page.php?pgcontent = mainPage) (Tautenhahn, Patti, Rinehart, & Siuzdak, 2012) based on default parameters. Finally, the metabolite annotation of GC‐MS was performed using the automatic processing and identification system (AMDIS) databases of the National Institute of Standards and Technology (NIST).

### 4.7 Quantitative real‐time PCR (qRT‐PCR)

To confirm the RNA‐Seq expression of the significant DEGs analyses, total RNAs were extracted from aerial and submerged mycelia following 5, 12 and 19 days of culture. The quantitative RT‐PCR was quantified using RNA‐Direct^TM^ SYBR green real‐time PCR master mix (Toyobo Co., LTD, Osaka, Japan) using the following conditions: 90°C: 30 s, 60°C: 20 min, 95°C: 1 min, 95°C: 15 s; 59°C: 15 s, and 74°C: 50 s for 40 cycles and performed in Mx3000P QPCR. Nine genes were selected and quantified using quantitative RT‐PCR (qRT‐PCR), including *alcohol dehydrogenase CCM_09633, alcohol dehydrogenase CCM_02484, hexokinase CCM_06280, aldehyde dehydrogenase CCM_02203, glucose‐6‐phosphate dehydrogenase CCM_06983, adenylate cyclase, putative CCM_02396, oxidoreductase CCM_ 01934, succinate dehydrogenase CCM_07146, *and* 5’‐nucleotidase CCM_00622*. The primer sets used are listed in Appendix Table A2. The relative gene expression was calculated using the 2^−ΔΔ^CT method (Livak & Schmittgen, 2001), and the *Rho GTPase activator *(Sac7) *CCM_07283* (Llanos, Francois, & Parrou, 2015) was used as a reference gene to quantify the relative expression levels of the nine genes.

## CONFLICT OF INTERESTS

The authors declare no conflict of interest.

## AUTHORS CONTRIBUTION

AS performed all the experiment, data analysis, and writing the draft manuscript. HT performed the GC‐MS and data curation. TK discussed about this work and gave adequate advice. EYP and TK revised draft manuscript. EYP supervised the laboratory work of AS.

## ETHICS STATEMENT

None required.

## DATA ACCESSIBILITY

All data are provided in full in the article, apart from the raw data of GC MS available in Supplementary Table S1 at [link to https://doi.org/10.6084/m9.figshare.7772903] and RNA sequence data, which is available in SRA [link to NCBI Sequence Read Archive (SRA)/BioProject accession number PRJNA524446].

## Figures and Tables

**Table A1:**
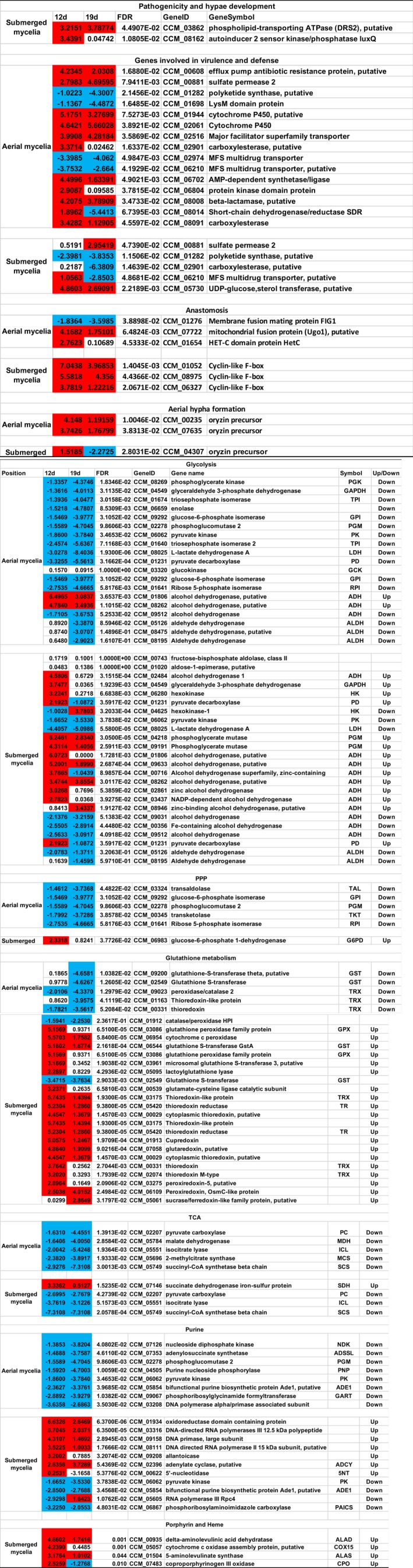
Summary of RNA‐seq analysis. Global DEGs between aerial mycelia and submerged mycelia. Red color represents the upregulated gene and blue color represents the downregulated gene

## APPENDIX 1

**Table A2:** List of primer that used for qRT‐PCR

No	Primer name	Sequence
1	Alcohol dehydrogenase XM_006674766 CCM_09633	F: GGTTCGTGGGCGAGTATCTG R: CCTGCGGATACACCACTAGC
2	Alcohol dehydrogenase 1 XM_006667636 CCM_02484	F: AGGAGAAGCCGTTTCAGCAG R: GCGGAAAAACTCAATGGCCT
3	Hexokinase XM_006671421 CCM_06280	F: CGCCCTCTAGAAAAGCCGAT R: GAAGTGCTCTGTGATGGCCT
4	Phosphoglycerate mutase XM_006669366 CCM_04218	F: AATGGGACTCTATGCGCGAG R: GCAGCATTATCTGTGCTGGC
5	Pyruvate decarboxylase XM_006666388 CCM_01231	F: ATTCCAACTCACGGCTCAGG R: CGACATGTTTGGCACACGTT
6	Aldehyde dehydrogenase XM_006667355 CCM_02203	F: CCGCCGTATACTAACGCCAA R: CAAGTCCTCCAGAGTCCAGC
7	Glucose‐6‐phosphate 1‐dehydrogenase (G6PD) XM_006672121 CCM_06983	F: CGATTGGAAGGAGGAGGAGC R: ACTCGTCAAAGTAGCCACCG
8	Rho GTPase activator (Sac7) XM_006672421 CCM_07283	F: CGAGAAGCGCATCAAAGAGC R: AAAGGAACGACAGGCTCAGG
9	Adenylate cyclase, putative XM_006667548.1 (CCM_02396)	F: CATGGTCGCACCGATGTAGA R: AAGAGCTATGCCAAAGGCGT
10	Oxidoreductase domain containing protein XM_006667088.1 (CCM_01934)	F: GTACTCCGACCGTGTCATCC R: TACCTTGTCCACATCGGTGC
11	Succinate dehydrogenase iron‐sulfur protein XM_006672284.1 (CCM_07146)	F: GAGTGCATTCTCTGCGCTTG R: TTGTTCTCGAGGTTGGCCTG
12	5’‐Nucleotidase XM_006665782.1 (CCM_00622)	F: GTTCTCTCCGAGGCCCTAGA R: AAACCCGGACGCGATAAAGT
